# Supplementary material for: Interpretation of biological experiments changes with evolution of the Gene Ontology and its annotations
Source: Sci Rep. 2018 Mar 23;8:5115. doi: 10.1038/s41598-018-23395-2 (PMC5865181; doi:10.1038/s41598-018-23395-2)
Supplement: Supplementary file 1 — Supplementary Information [file 41598_2018_23395_MOESM1_ESM.pdf]

Supplementary Information - Interpretation of biological experiments changes with evolution of Gene Ontology and its annotations

Aurelie Tomczak[1], Jonathan M. Mortensen[2], Rainer Winnenburg[2], Charles Liu[1], Dominique T. Alessi[2], Varsha Swamy[1], Francesco Vallania[1], Shane Lofgren[1], Winston Haynes[1,2], Nigam H. Shah[2], Mark A. Musen[2], Purvesh Khatri[1,2,*]

[1]Stanford Institute for Immunity, Transplantation and Infection (ITI), Stanford University, Stanford, CA 94305

[2]Stanford Center for Biomedical Informatics Research (BMIR), Department of Medicine, Stanford University, Stanford, CA 94305

* To whom correspondence should be addressed. Tel: (650) 497 5281; Fax: 650 725 7944; Email: pkhatri@stanford.edu

Supplementary Dataset 1

Supplementary data table 1: significantly differentially expressed transcript IDs at FDR 0.01

- 1,003 gene/transcript IDs (mapping to 967 genes) for influenza,
- 3,677 gene/transcript IDs for non-small cell lung cancer and
- 3,468 gene/transcript IDs for pancreatic cancer

Supplementary data table 2:

- All significant GO terms for influenza, pancreatic and non-small cell lung cancer for yearly GO updates (year of ontology version = year of GO annotation version, p-value < 0.05)

Supplementary data table 3:

- List of all multi-cohort analyses of diseases analyzed in this study with GEO datasets used

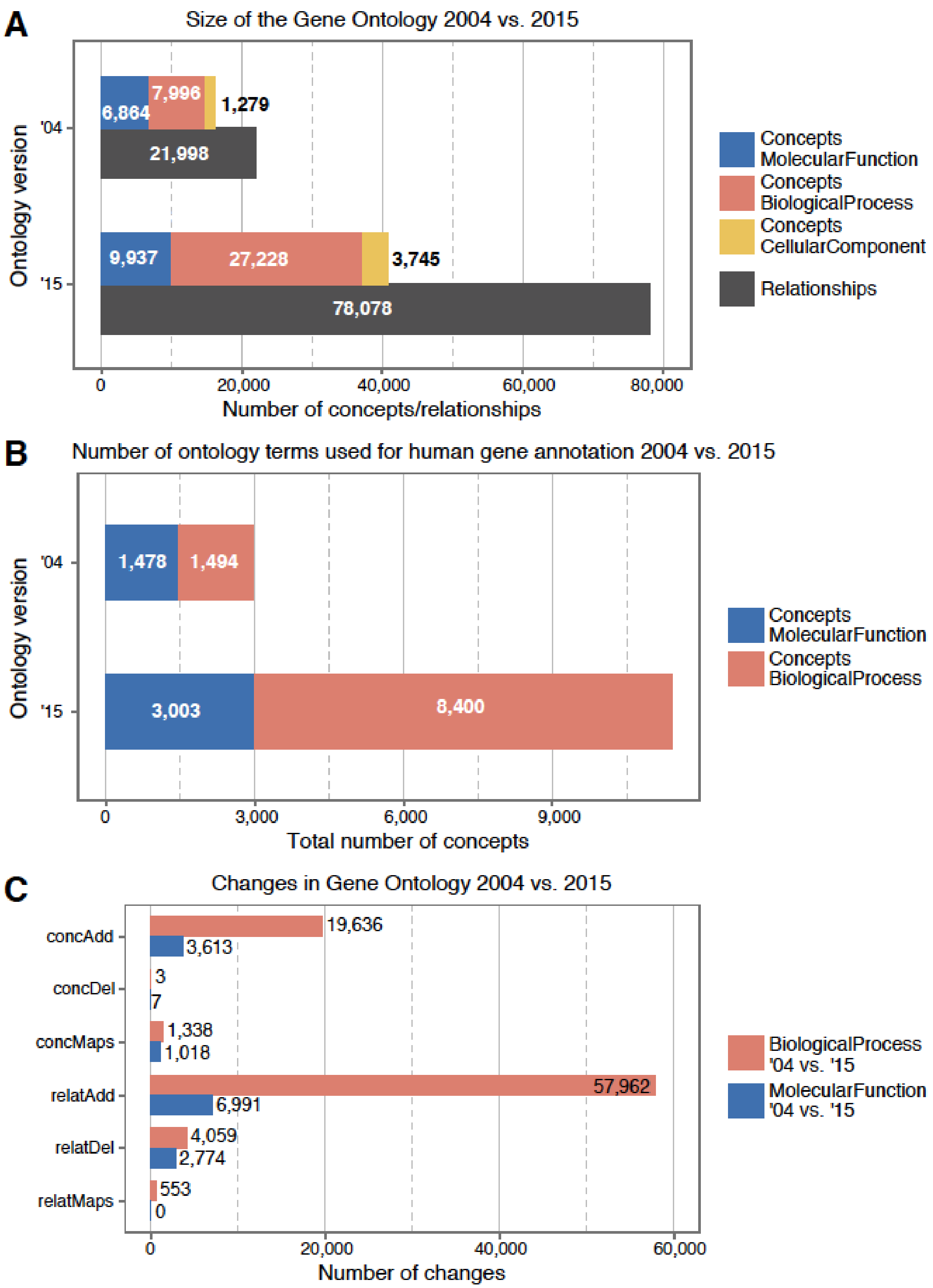

Supplementary Figure 1: Gene ontology and annotation developments from 2004 to 2015. A) Growth in the number of GO terms/concepts in three ontologies: biological process (BP), molecular function (MF), and cellular component (CC), and relationships between them. **B)** Number of BP and MF ontology concepts used for annotation of human genes 2004 and 2015. **C)** Changes in BP and MF ontologies between 2004 and 2015 ('conc': concept/term, 'relat': relationship, 'Add': additions, 'Del': deletions, 'Maps': mappings).

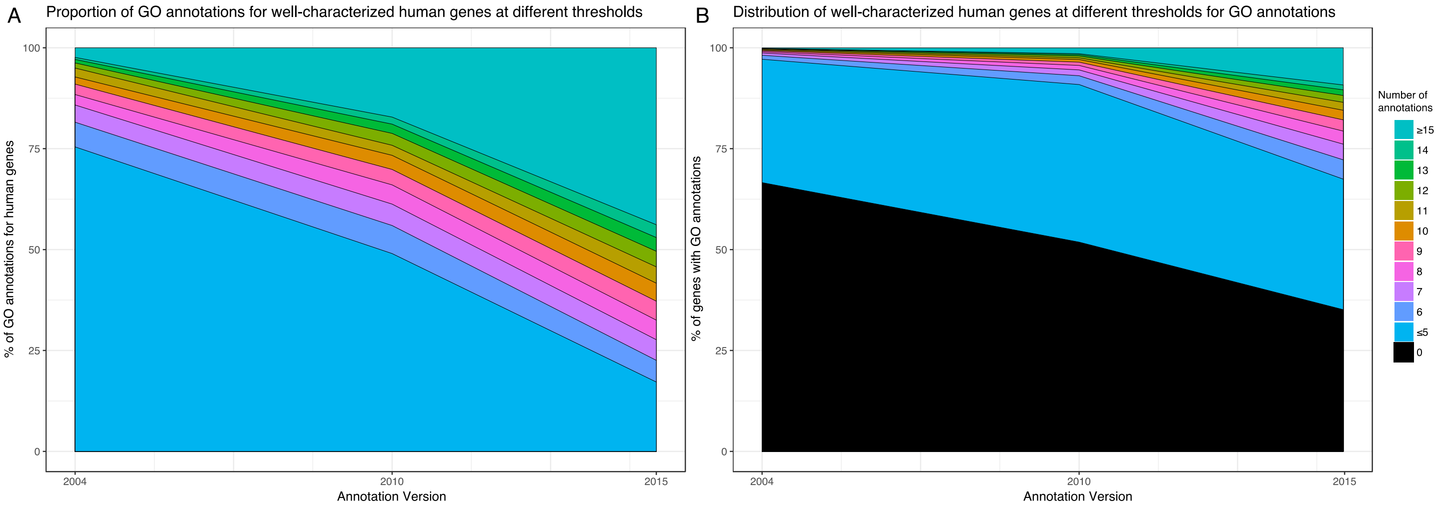

Supplementary Figure 2: Change in the Gene ontology annotations for human genes grouped by number of GO annotations per gene. A) Proportion of GO annotations and their distribution across human genes grouped by number of annotations per gene over time. **B)** GO annotation status of the human genome (2004 vs. 2015). Genes are grouped by annotation status in uncharacterized (black) vs. characterized (colored) and grouped by number of annotations per gene. Only terms relevant for enrichment analysis results were counted (excluding: IEA, ND and cellular component).

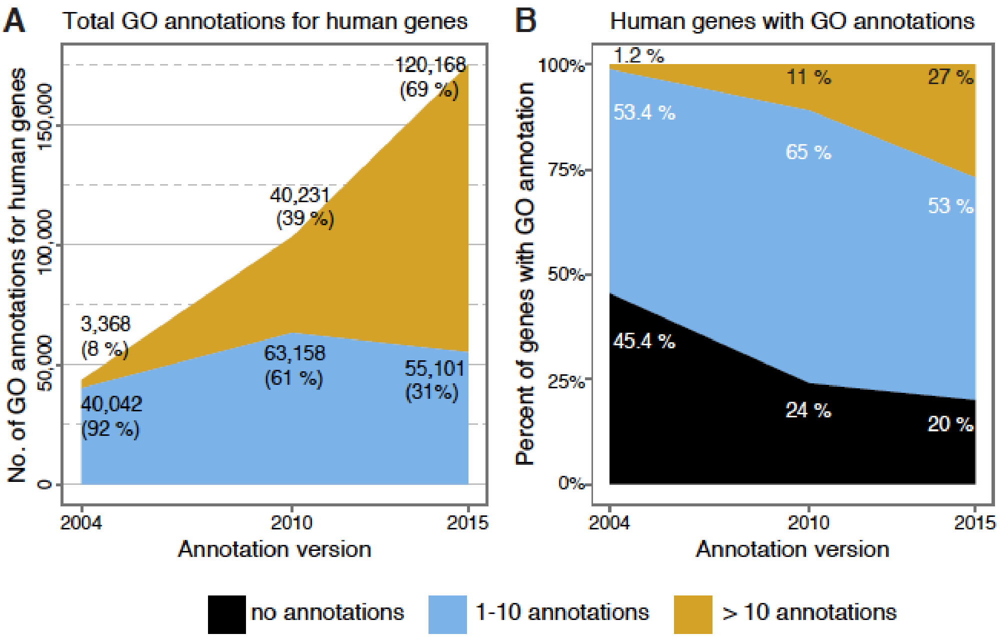

Supplementary Figure 3: Gene ontology annotation developments including electronic annotations, human genome, 2004 to 2015. A) Number and distribution of GO annotations across poorly characterized (blue) vs. well-characterized human genes (gold) over time. **B)** GO annotation status of the human genome (2004 vs. 2015). Genes are classified by annotation status in uncharacterized (black) vs. poorly characterized (blue) vs. well characterized (gold). Only terms relevant for enrichment analysis results were counted (excluding ND and cellular component).

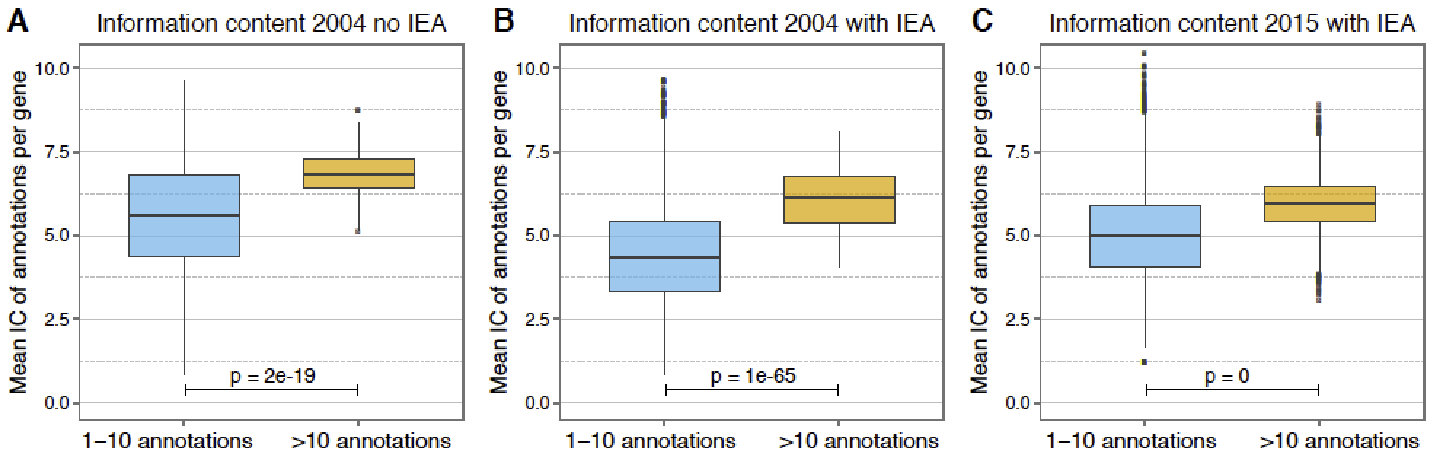

Supplementary Figure 4: Mean Information Content. Comparison of mean information content (IC) of poorly characterized (blue) vs. well-characterized human genes (gold) shows that the mean IC for genes with more annotations was higher, independently of GO version and electronic annotations (evidence code: IEA). **A)** Mean IC 2004 without IEA, **B**) 2004 with IEA and **C)** 2015 with IEA.

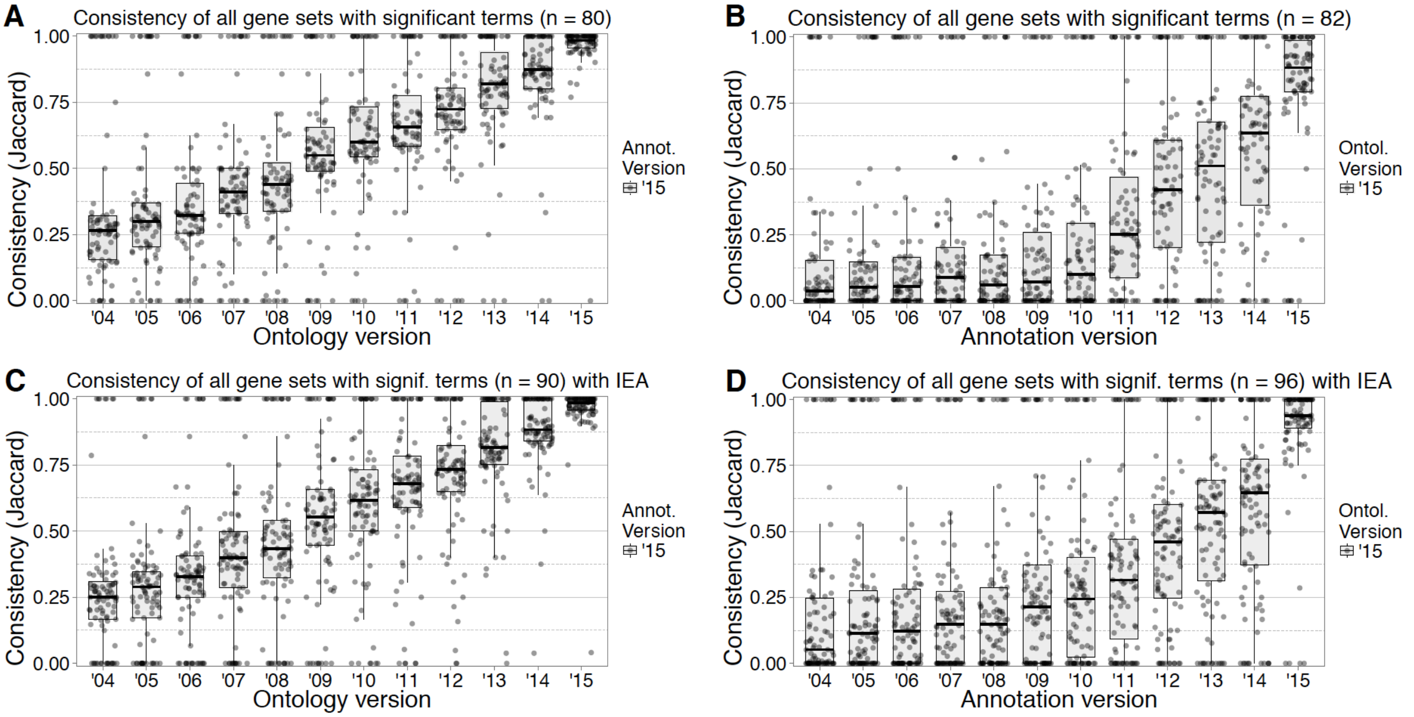

Supplementary Figure 5: Effect of ontology and annotation version on consistency and significance of GO enrichment analysis results. A) Consistency (Jaccard index) for n gene sets with at least one significant term (80 gene sets) with changing ontology versions (annotation version: January 2015 annotations), and **B)** with changing annotation version (ontology version: January 2015 ontology, n=82 disease gene sets, reference version for comparison: March 2015 ontology and annotations). **C)** Consistency differences for 90 gene sets with at least one significant term with changing ontology versions (annotation version: January 2015 annotations) including electronic annotations, and **D)** with changing annotation version (ontology version: January 2015 ontology, n = 96 gene sets, reference for comparison: March 2015 ontology and annotations).

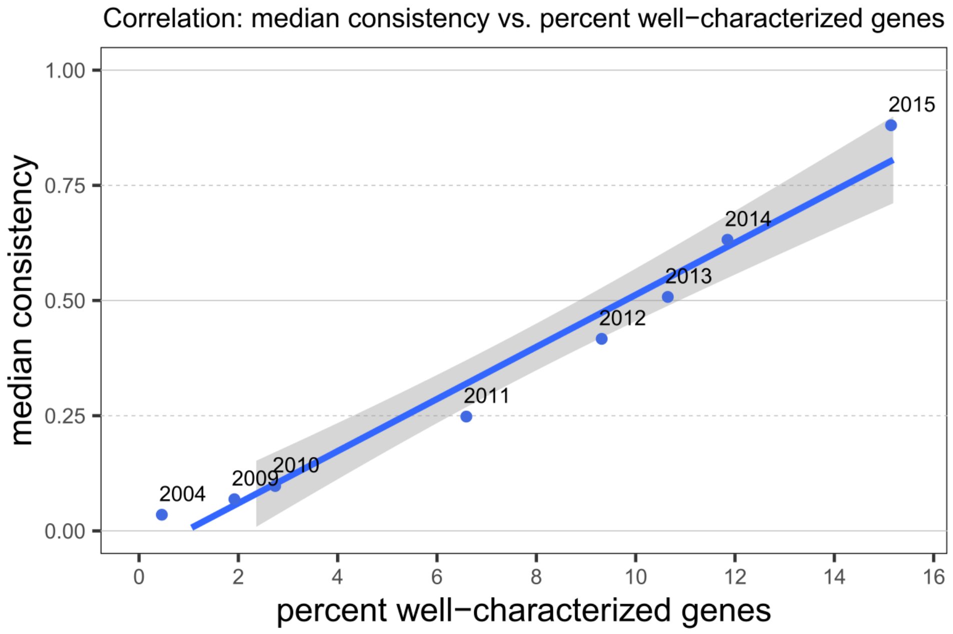

Supplementary Figure 6: Correlation of median consistency vs. percent of well-characterized genes over time. High correlation is observed (Pearson correlation coefficient: 0.984) when median consistency with changing annotation versions and fixed ontology (Supp. Fig. 4B) is compared to percentages of well-characterized genes per year for 2004 and between 2009 and 2015.

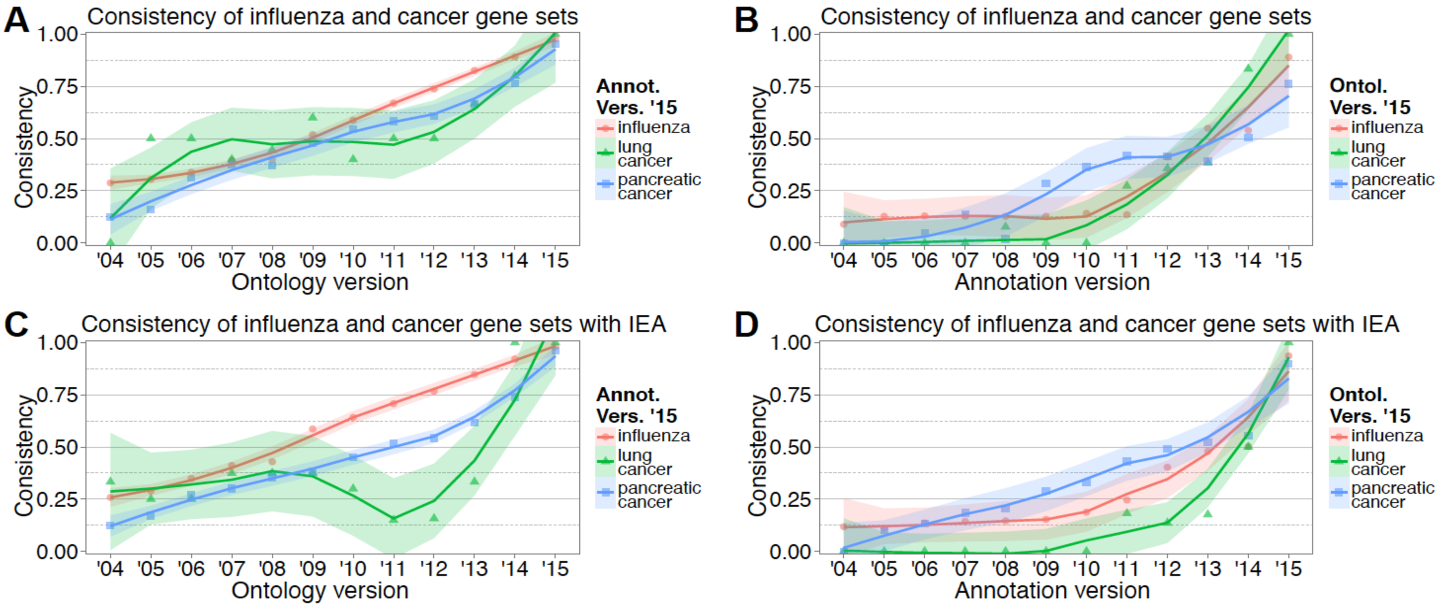

Supplementary Figure 7: Effect of ontology and annotation version on consistency and significance of GO enrichment analysis results in individual diseases. Variation in enrichment analysis consistency (Jaccard index) for disease gene sets for influenza, lung cancer, and pancreatic cancer with **A)** different ontology versions and **B)** different annotation versions. Variation of consistency after including electronic annotations for disease gene sets with **C)** different ontology versions and **D)** with different annotation versions.

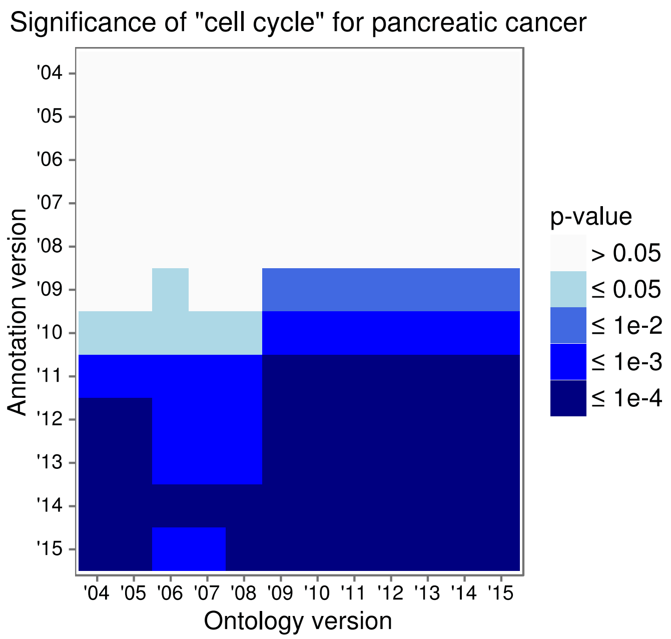

Supplementary Figure 8: GO term enrichment significance for cell cycle in pancreatic cancer.

Supplementary Table

Number of annotations (X)	0	1	2	3	4	5	6	7	8	9	10	11	12	13	14	>=15
Year	Percentages of total human genes with X annotations															
2004	66.6%	8.0%	10.4%	6.9%	3.7%	1.6%	1.0%	0.6%	0.31%	0.27%	0.17%	0.19%	0.10%	0.06%	0.03%	0.13%
2010	51.9%	12.9%	10.5%	7.6%	4.8%	3.3%	2.2%	1.4%	1.1%	0.8%	0.7%	0.42%	0.46%	0.33%	0.23%	1.4%
2015	35.1%	7.2%	7.2%	6.9%	5.8%	5.2%	4.8%	3.9%	3.2%	2.8%	2.4%	2.0%	1.7%	1.4%	1.2%	9.2%
Year	Percentages of total GO annotations describing genes with X annotations															
2004	0	8.4%	21.6%	21.7%	15.3%	8.5%	6.1%	4.2%	2.6%	2.6%	1.8%	2.2%	1.3%	0.9%	0.5%	2.4%
2010	0	6.8%	11.1%	12.2%	10.2%	8.6%	6.9%	5.3%	4.8%	3.8%	3.5%	2.5%	3.0%	2.3%	1.7%	17.2%
2015	0	1.3%	2.7%	3.9%	4.4%	4.9%	5.4%	5.1%	4.8%	4.7%	4.5%	4.0%	3.9%	3.4%	3.2%	43.8%

Supplementary Table 1: Distribution of GO annotations across the human genome.
